# Pim1 Kinase Inhibitors Exert Anti-Cancer Activity Against HER2-Positive Breast Cancer Cells Through Downregulation of HER2

**DOI:** 10.3389/fphar.2021.614673

**Published:** 2021-06-29

**Authors:** Bo-Wei Wang, Chih-Hao Huang, Liang-Chih Liu, Fang-Ju Cheng, Ya-Ling Wei, Yueh-Ming Lin, Yu-Fei Wang, Ching-Ting Wei, Yeh Chen, Yun-Ju Chen, Wei-Chien Huang

**Affiliations:** ^1^ Graduate Institute of Biomedical Sciences, Center for Molecular Medicine and Research Center for Cancer Biology, China Medical University, Taichung, Taiwan; ^2^ Division of Breast Surgery, China Medical University Hospital, Taichung, Taiwan; ^3^ Division of Colorectal Surgery, Department of Surgery, Kaohsiung Chang Gung Memorial Hospital and Chang Gung University College of Medicine, Kaohsiung, Taiwan; ^4^ School of Medicine for International Students, I-Shou University, Kaohsiung, Taiwan; ^5^ Institute of New Drug Development, China Medical University, Taichung, Taiwan; ^6^ Department of Medical Research, E-Da Hospital, Kaohsiung, Taiwan; ^7^ Drug Development Center, China Medical University, Taichung, Taiwan; ^8^ Department of Medical Laboratory Science and Biotechnology, Asia University, Taichung, Taiwan; ^9^ The Ph.D. Program for Cancer Biology and Drug Discovery, China Medical University and Academia Sinica, Taichung, Taiwan

**Keywords:** HER2, lapatinib, drug resistance, breast cancer, PIM1

## Abstract

The proviral integration site for moloney murine leukemia virus 1 (Pim1) is a serine/threonine kinase and able to promote cell proliferation, survival and drug resistance. Overexpression of Pim1 has been observed in many cancer types and is associated with the poor prognosis of breast cancer. However, it remains unclear whether Pim1 kinase is a potential therapeutic target for breast cancer patients. In this study, we found that Pim1 expression was strongly associated with HER2 expression and that HER2-overexpressing breast cancer cells were more sensitive to Pim1 inhibitor-induced inhibitions of cell viability and metastatic ability. Mechanistically, Pim1 inhibitor suppressed the expression of HER2 at least in part through transcriptional level. More importantly, Pim1 inhibitor overcame the resistance of breast cancer cells to HER2 tyrosine kinase inhibitor lapatinib. In summary, downregulation of HER2 by targeting Pim1 may be a promising and effective therapeutic approach not only for anti-cancer growth but also for circumventing lapatinib resistance in HER2-positive breast cancer patients.

## Introduction

Breast cancer is the most common cancer type and ranks second among causes for cancer death in women (Fahad Ullah, 2019). According to the expression pattern of biomarkers, including estrogen receptor (ER), progesterone receptor (PR) and human epidermal growth factor receptor 2 (HER2, also known as Neu, ErbB2, EGFR2), breast cancer can be classified into several subtypes (Raica et al., 2009). Among these biomarkers, HER2 overexpression is correlated with poor prognosis prior to the advent of anti-HER2 therapies (Barros et al., 2010; Santa-Maria et al., 2016).

HER2 is a member of human epidermal growth factor receptor (HER/EGFR) tyrosine kinase family, which is frequently overexpressed in many cancer types (Wang, 2017). HER family includes EGFR, HER2, HER3, and HER4. The overexpressed HER2 form either homo-dimer or hetero-dimer with other members of EGFR family. Thereafter, HER2 is activated through autophosphorylation and transduces the downstream signaling pathways, leading to cycle progression, cell proliferation, survival and cancer stemness for tumor progression (Hsu and Hung, 2016; Nami and Wang, 2017). Therefore, targeted therapy against HER2 tyrosine kinase activity has been developed and approved for HER2-positive breast cancer (Riese and Stern, 1998; Iqbal and Iqbal, 2014). There are two types of HER2-targeted therapy, including HER2 antibody trastuzumab and HER2 tyrosine kinase inhibitor (TKI) lapatinib (Ahmed et al., 2015). Furthermore, lapatinib may act as a surrogate treatment for HER2-overexpressing metastatic breast cancer patients who failed to respond to trastuzumab treatment (Brandes et al., 2010; Hicks et al., 2015). Although these drugs indeed show clinical benefits to HER2-positive breast cancer patients, acquired resistance is still developed eventually and remains a hurdle to be overcome (Nahta et al., 2009; Rexer and Arteaga, 2012; Pernas and Tolaney, 2019). However, the mechanisms underlying resistance remain not fully clarified.

The proviral integration site for moloney murine leukemia virus 1 (Pim1) is a serine/threonine kinase. There are three members in human Pim family, including Pim1, Pim2, and Pim3, which are encoded in chromosome 6, X chromosome, and chromosome 22, respectively. Aberrant elevation of Pim1 has been observed in many cancer types and reported to play a crucial role in tumorigenesis due to the interactions with numerous proteins participating in various signaling pathways involved in cell proliferation, survival, and drug resistance (Narlik-Grassow et al., 2014; Warfel and Kraft, 2015). The oncogenic potential of Pim1 was most extensively investigated in prostate cancer (Holder and Abdulkadir, 2014; Ouhtit et al., 2015; Luszczak et al., 2020). It has reported that AKT inhibitor GSK690693 promotes the transcriptional induction of Pim1 kinase, which increased the protein expressions of receptor tyrosine kinase (RTK), including EGFR, HER2, and HER3, and subsequently resulted in the resistance of prostate cancer cells to AKT inhibition (Cen et al., 2013). Furthermore, Pim kinase inhibitor M-110 was shown to reduce the expression of EGFR, leading to the reduction of extracellular signal-regulated kinase (ERK) pathway activity in prostate cancer (Siu et al., 2011). Although overactivation of HER family was observed in many cancers, especially in breast cancer, ovarian cancer, and non-small cell lung cancer, and correlates with poor prognosis and drug resistance (Wang, 2017), it remains unclear whether Pim1 plays a role in the regulations of HER family expression and TKI resistance and functions a potential therapeutic target in breast cancer. In this study, our data showed that Pim1 positively regulates the expressions of HER2 at the transcriptional level and that targeting Pim1 may be a promising and effective therapeutic approach not only for anti-cancer growth but also for circumventing lapatinib resistance in HER2-positive breast cancer patients.

## Materials and Methods

### Cell Lines and Cell Culture

Human HER2-positive (SkBr3, BT474) and HER2-negative (MDA-MB-231, MCF7, and T47D) breast cancer cell lines were obtained from the American Type Culture Collection. HBL-100 cells and HER2-overexpressing clone (HER18) of MCF-7 cells were 5kind gift from Prof. Mien-Chie Hung. Lapatinib-resistant clones (Sk/LR6 and Sk/LR9) were selected from SkBr3 cells by culturing the cells in increasing concentrations of lapatinib (by 2 µM every 2–3 weeks, up to a maintenance concentration of 10 µM for 3 months). All cell lines were maintained in Dulbecco’s Modified Eagle’s Medium/F12 containing 10% fetal bovine serum (GeneDireX), 100 U/ml penicillin, and 100 μg/ml streptomycin (Thermo Fisher Scientific) and incubated at 37°C in a humidified atmosphere of 95% air and 5% CO_2_. Lapatinib-resistant clones were maintained in the presence of 1 μM lapatinib.

### Preparation of Cell Extracts

Cells were washed with 1X phosphate buffered saline (PBS) once and harvested with RIPA buffer (50 mM Tris (pH7.5), 150 mM NaCl, 10 mM EDTA, 1% NP-40, 0.1% SDS, 1 mM PMSF, 10 μg/ml Aprotinin) plus protease inhibitors, followed by homogenization with sonication and centrifugation at 21,500 × g for 15 min. Whole cell lysates were stored at -20°C until used for the experiments (Lee et al., 2019a; Lee et al., 2020).

### Western Blot and Antibodies

As described previously (Lee et al., 2019b), the concentration of total proteins was determined by Bradford protein assay (Bio-Rad), and protein levels were examined by western blot analysis with specific antibodies. Antibody against p-Pim1 Tyr309 was purchased from Assay biotech. Antibodies against AKT, p-ERK Thr202/Tyr 204 and ERK were purchased from Cell Signaling. Antibodies against Pim1 (12H8), EGFR, HER3 (C-17), and HER4 (C-18) were purchased from Santa Cruz. Antibodies against α-Tubulin, Flag®M2 and β-Actin were purchased from SIGMA. Antibody against HER2 was purchased from EMD Millipore. Relative protein expressions were quantified by using ImageJ software (Wayne Rasband, National Institute of Health, United States ). The quantification was shown as the relative amounts of each protein normalization with the loading control, and data were represented for three independent experiments.

### 3-(4,5-Dimethylthiazol-2-yl)-2,5- Diphenyltetrazolium Bromide Assay

Cells were seeded at a density of 3–4 × 10^3^ cells/well in a 96-well plate. The next day, cells were cultured with serum-free medium and treated with Pim inhibitors SMI-4a and SGI-1776 at the concentrations of 0, 0.5, 1, 2, 5, 10, 20 μM for 2 days in three independent experiments. Then, the culture medium was refreshed with 100 μl serum-free medium with 5 mg/ml MTT solution (Sigma) for 3 h followed by wash with PBS 3 times. The formazan in the cells was solubilized in 100 μl DMSO per well, followed by the measurement of absorbance at 570 nm.

### Lentivirus Infection of shRNA

Cells were seeded at a density of 2 × 10^5^ cells/well in a 6-well plate overnight. Cells were infected with lentivirus shRNA using a multiplicity of infection of 125 for 24 h. Then, cells were refreshed with the medium containing 2 μg/ml puromycin for 24 h followed by subsequent experiments.

### Plasmid DNA Transfection

Cells were seeded at a density of 4 × 10^5^ cells/well in a 6-well plate. The next day, the cells were transfected with 1 μg plasmid DNA per well for 2 days using TransIT-2020 transfection reagent according to the manufacturer’s instruction as described previously (Huang et al., 2013), followed by subsequent experiments.

### Cell Migration and Invasion Assays

Cell migration and invasion abilities were examined by *in vitro* transwell assay as described previously (Huang et al., 2016). For migration assay, cells at a density of 2 × 10^5^/well were seeded on the non-coated membrane of the upper chamber. For invasion assay, the membrane of the upper chamber was coated with 1–2 mg/ml Matrigel (BD Biosciences), followed by cell seeding at a density of 2 × 10^5^/well with treatment of SMI-4a at the indicated concentration. After 48 h incubation, cells were washed with 1X PBS once, followed by fixation with 4% formaldehyde for 30 min. Cells were washed with 1X PBS once again, followed by 1% crystal violet staining for 15–30 min at room temperature. Cells remaining on the upper chamber were removed using cotton swab. The number of migrating or invading cells was shown and quantified by counting for five fields/field of view at ×200 magnification.

### Clonogenic Assay

HER2-negative and -positive breast cancer cells were seeded at a density of 1 × 10^3^/well in a 24-well plate. The next day, cells were treated with SMI-4a for 14 days. The cells were refreshed with a medium containing SMI-4a every 7 days. 2 weeks later, the cell viability was determined by 1% crystal violet staining (buffered with 30% ethanol).

### Reverse-Transcription-Quantitative Polymerase Chain Reaction

Total RNA extraction was performed using Trizol^™^ reagent (Roche). 1 μg RNA was subjected to reverse transcription using M-MLV reverse transcriptase according to manufacturer’s instruction (Sigma). The qPCR analysis was performed on Illumina EcoTM system (Bio-genesis Technologies Inc.) using VeriQuest Fast SYBR Green qPCR Master Mix.

### Determination of the Half-Maximal Inhibitory Concentration

IC50 of Pim inhibitors was determined by the following equation: lgIC50 = Xm-I (P- (3-Pm-Pn)/4). Xm: lg maximum dose; I: lg (maximum dose/relative dose); *p*: the sum of the positive reaction rate; Pm: the maximum positive reaction rate; Pn: the minimum positive reaction rate.

### Statistical Analysis

Pearson correlation was used to study the correlation between IC50, Pim1, or HER family expression in breast cancer cell lines. All data were displayed as mean ± S.E.M for three independent experiments. The significance of the difference between the experimental and control groups was assessed by Student’s t-test. The difference is significant if *p*-value is *< 0.05, **<0.01, ***<0.001.

## Results

### Human epidermal growth factor receptor 2 Expression Was Strongly Associated With the Expression and Inhibitor Sensitivity of Pim1

**FIGURE 1:**
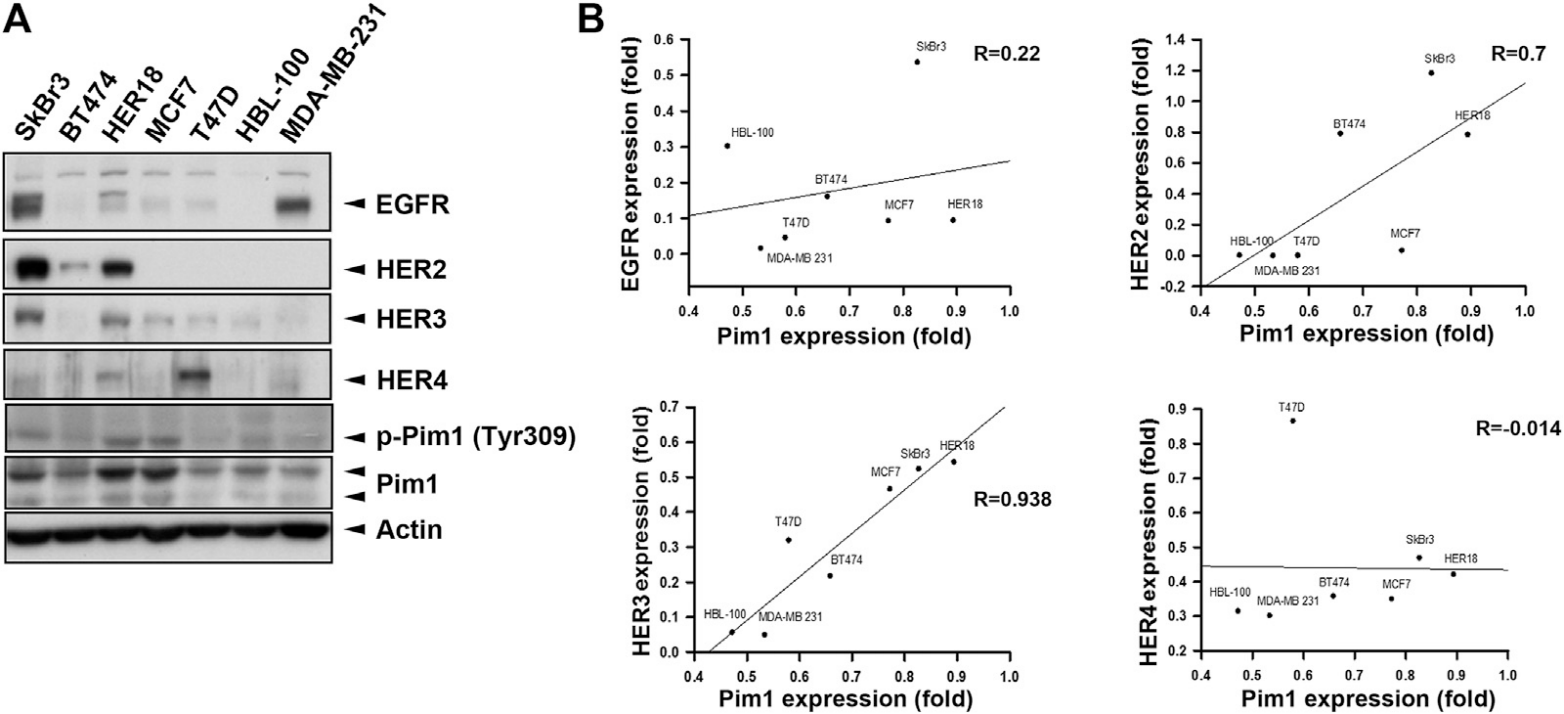
HER2 expression was strongly associated with the Pim1 expression in breast cancer cells. **(A)** Whole cell lysates of breast cancer cells, including SkBr3, BT474, HER18, MCF7, T47D, HBL-100, and MDA-MB-231 cells, were subjected to protein expression analysis in western blot using the indicated antibodies. **(B)** The correlations of Pim1 expression with EGFR, HER2, HER3, and HER4 expression were analyzed by regression analysis based on the results shown in **(A)**.

It is known that induction of Pim1 was accompanied by increases in EGFR expression (Siu et al., 2011; Cen et al., 2013). To address whether Pim1 regulates HER family expression in breast cancer, we first examined the association between Pim1 and HER family protein expressions using a panel of breast cancer cell lines by western blot (Figure 1A). The correlation analysis based on *R* ^2^ score revealed that Pim1 protein expression significantly and positively correlated with HER2 and HER3, but not HER4, protein expressions and that the correlation between Pim1 and EGFR expressions approaches marginal significance (Figure 1B).

**FIGURE 2:**
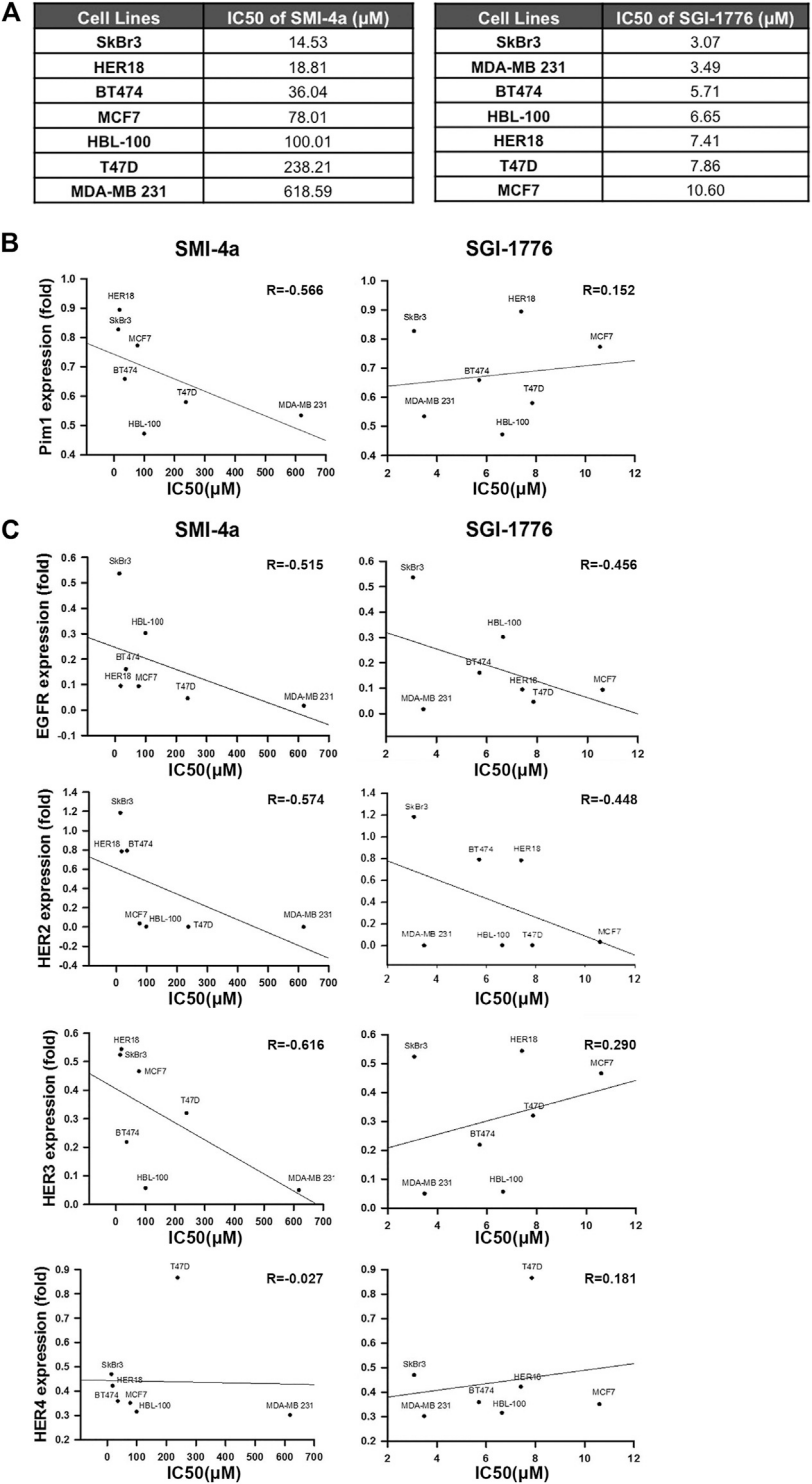
HER2 expression was strongly associated with the sensitivity of breast cancer cells to Pim1 inhibitors**. (A)** The summary table of IC50 of Pim1 inhibitors in various breast cancer cell lines. **(B,C)** The half-maximal inhibitory concentration (IC50) of Pim1 inhibitors, including SMI-4a and SGI-1776 in SkBr3, BT474, HER18, MCF7, T47D, HBL-100, and MDA-MB-231 cells, was determined by MTT assays. The correlations of IC50 of SMI-4a and SGI-1776 with Pim1 **(B)**, EGFR **(C)**, HER2 **(C)**, HER3 **(C)** and HER4 **(C)** expressions were determined by regression analysis.

To further address whether the correlation between Pim1 and HER family expressions relies on Pim1 kinase activity, Pim1 kinase inhibitors SMI-4a and SGI-1776 were employed. First, we determined the sensitivity of various breast cancer cell lines to these Pim1 inhibitors in MTT assays and analyzed the correlation of Pim1 protein expression with the IC50 of these two inhibitors. The IC50 of these Pim1 inhibitors in various breast cancer cell lines were listed in Figure 2A. Alteration of protein level is one of the factors contributing to oncogenic function and may determine the sensitivity of cancer cells to their inhibitors, and the target-independent cell-killing effect of SGI-1776 has been reported (Lin et al., 2019). Therefore, we first analyzed the correlation of Pim1 protein expression with the IC50 of these two inhibitors. As shown in Figure 2B, the IC50 of SMI-4a but not SGI-1776 was inversely associated with Pim1 protein expression, indicating that the specific inhibition of Pim1 by SGI-1776 is not the sole mechanism for its anti-cancer activities. We next analyzed the correlation between HER family protein levels and the IC50 of these two inhibitors. We found that the IC50 of SMI-4a significantly and inversely correlated with EGFR, HER2, and HER3 protein levels while the IC50 of SGI-1776 only significantly and negatively correlated with EGFR and HER2 protein level (Figure 2C). Taken together, these results suggest that EGFR and HER2 expressions are strongly associated with Pim1 expression and the sensitivity to Pim1 inhibitors in breast cancer cells.

### Human epidermal growth factor receptor 2 -Expressing Breast Cancer Cells Were More Sensitive to Pim1 Inhibitor-Induced Cell Death

**FIGURE 3:**
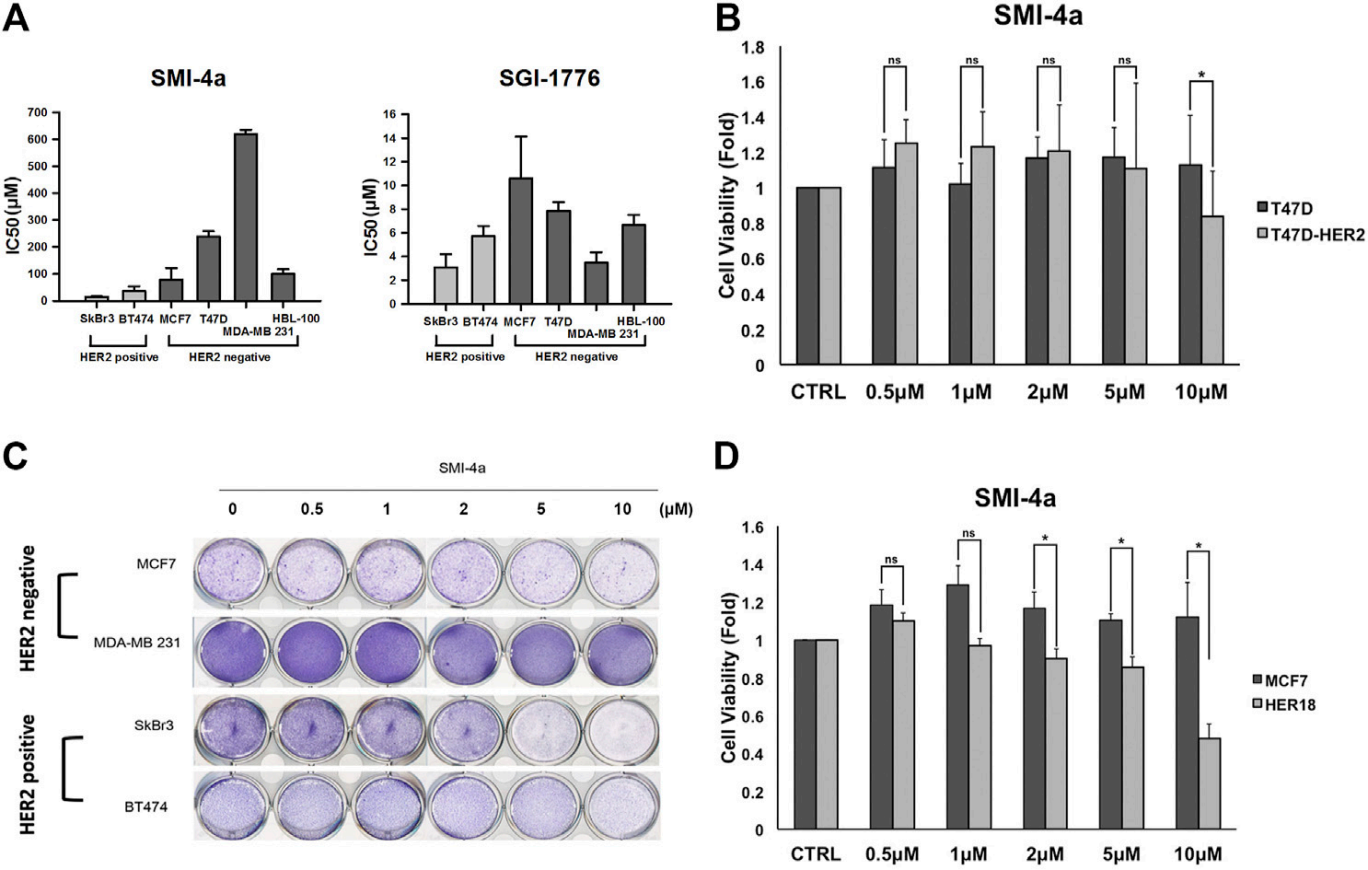
HER2-expressing breast cancer cells were more sensitive to Pim1 inhibitor-induced inhibition of cell viability. **(A)** The IC50 of SMI-4a and SGI-1776 in SkBr3, BT474, MCF7, T47D, HBL-100, and MDA-MB-231 cells was determined by MTT assay. **(B)** HER2 expression vector or empty vector was transiently transfected into HER2-positive SkBr3 or BT474 cells for 2 days, followed by the determination of cell viability in response to SMI-4a in MTT assay. **(C)** The effects of SMI-4a on the cell viabilities of MCF7, HER18, and HER2-transfected T47D cells were determined in MTT assay. Results were expressed as mean ± S.E.M. of three independent experiments. *: *p* < 0.05; **: *p* < 0.01 as compared with control group. **(D)** MCF7, HER18, T47D and T47D-HER2 cells were treated with SMI-4a at 10 μM and subjected to clonogenic assay for 14 days. Cell viability was determined by crystal violet staining.

**FIGURE 4:**
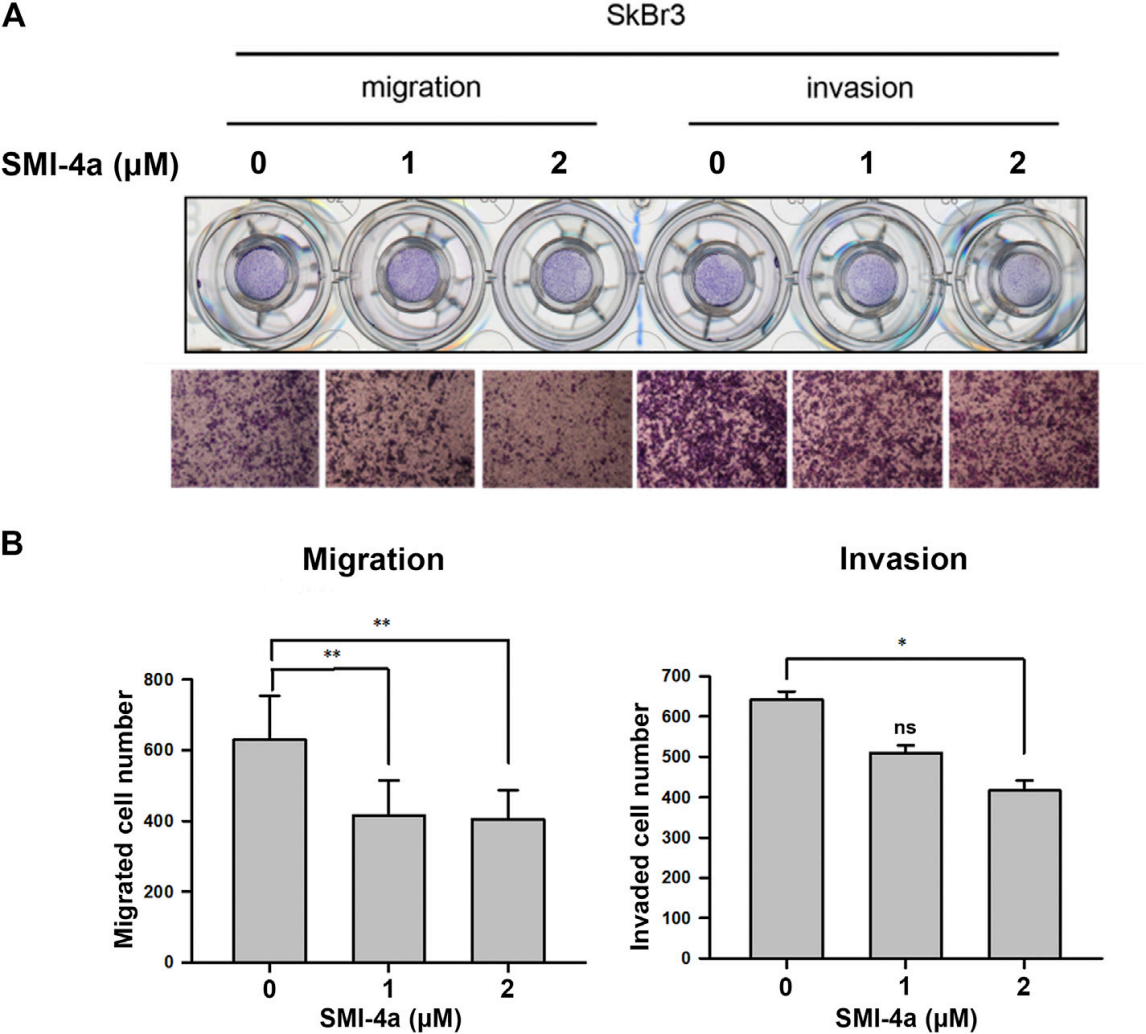
Pim1 inhibitor attenuated cell migration and invasion abilities in HER2-positive SkBr3 cells. **(A,B)** SkBr3 cells were treated with SMI-4a at 0, 1, 2 μM for 2 days and subjected to *in vitro* transwell assay. Cell migration and invasion were observed under microscope and by crystal violet staining **(A)**. The numbers of migrated and invaded cells were calculated and quantified **(B)**. Results were expressed as mean ± S.E.M. of three independent experiments. *: *p* < 0.05; **: *p* < 0.01 as compared with control group.

Since we observed that HER2 expression was associated with Pim1 inhibitor sensitivity, we further examine whether HER2-positive breast cancers are more sensitive to Pim1 inhibitors. As shown in Figure 3A, IC50 values of SMI-4a and SGI-1776 were lower in HER2-positive than in HER2-negative breast cancer cell lines. In order to investigate whether HER2 acts as a determinant for the sensitivity to Pim1 inhibitors, HER2 was overexpressed in different breast cancer cells followed by measuring their sensitivity to SMI-4a. The viabilities of HER2-addicted SkBr3 and BT474 breast cancer cells were suppressed by SMI-4a, and the inhibitory effect was rescued by further increasing HER2 expression in these cell lines (Figure 3B). On the other hand, HER2-negative and Pim1 inhibitor-insensitive MCF7 and T47D cells became sensitive to SMI-4a while these cells were transformed to HER2-positive and addicted (HER18 and T47D-HER2) cells in MTT assay (Figure 3C). Similar results were also observed in clonogenic assays (Figure 3D, Supplementary Figure S1). In addition to cell viability, we also examined the effect of the Pim1 inhibitor on cell migration and invasion. As shown in Figure 4A, SMI-4a reduced the migration and invasion abilities of HER2-positive SkBr3 breast cancer cells in a dose-dependent manner. The quantitative results of migrated and invaded cell numbers were shown in Figure 4B. Collectively, these findings support that HER2 acts as one of the Pim1 downstream effectors and is a critical determinant for the sensitivity of HER2-positive cells to Pim-1 inhibitor. However, the possibility that other downstream effectors of Pim1 mediate the anti-cancer activity of Pim1 inhibitor in HER2-positive breast cancer cells can not be excluded.

### Pim1 Inhibitors Suppressed Human epidermal growth factor receptor Family Expression in Human epidermal growth factor receptor 2 -Expressing Breast Cancer Cells

**FIGURE 5:**
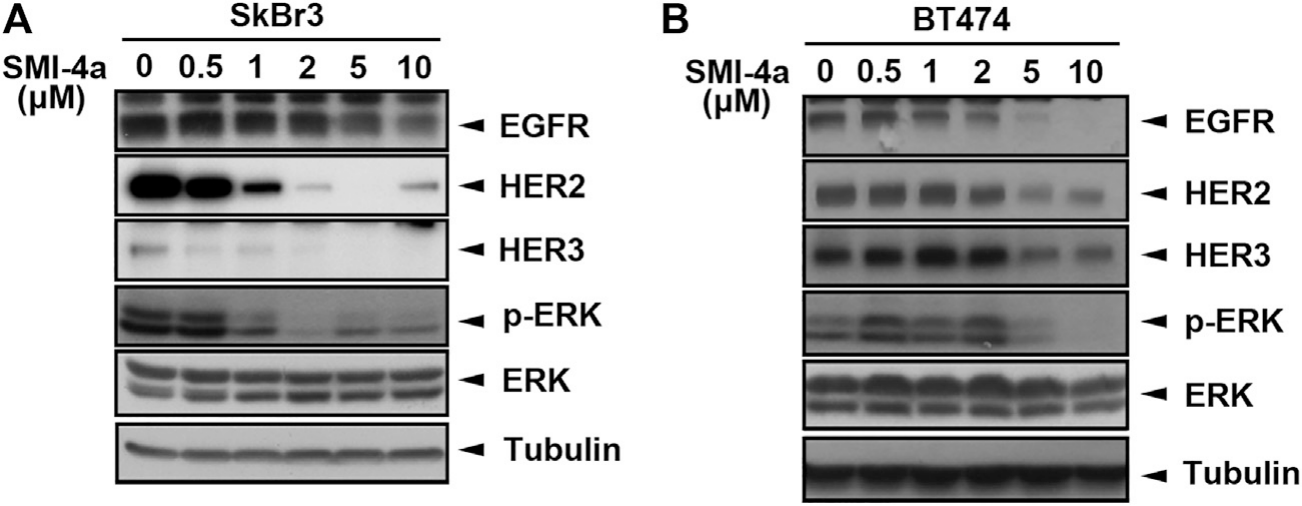
Pim1 inhibitors suppressed HER family expression in HER2-expressing breast cancer cells. **(A,B)** SkBr3 **(A)** and BT474 **(B)** cells were treated with SMI-4a at 0, 0.5, 1, 2, 5, 10 μM, and whole-cell lysates were harvested. Protein expressions were examined by western blot using the indicated antibodies.

**FIGURE 6:**
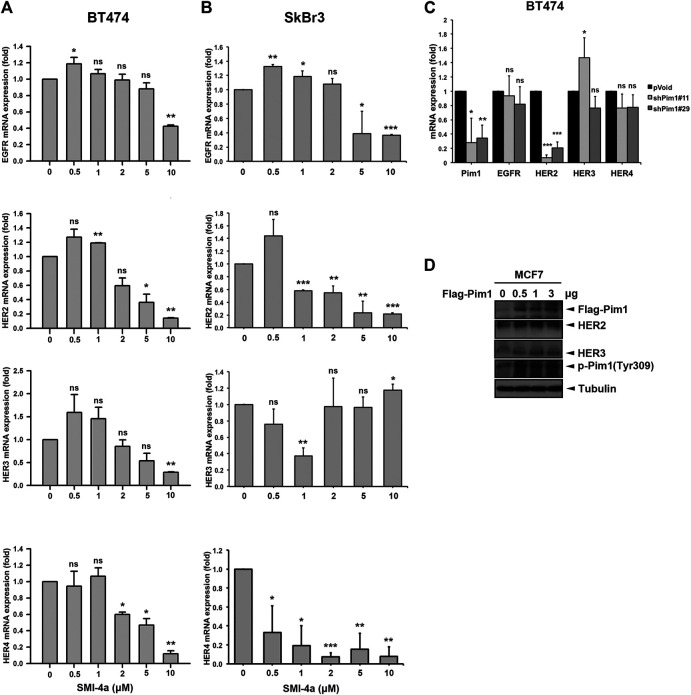
Pim1 transcriptionally upregulated HER2 expression in breast cancer cells. **(A,B)** BT474 **(A)** and SkBr3 **(B)** cells were treated with SMI-4a at 0, 0.5, 1, 2, 5, 10 μM and total RNAs were extracted. The mRNA expressions of the HER family were examined by RT-qPCR followed by normalization with *actin* expression. **(C)** BT474 cells were infected with shPim1#11 or shPim1#18 lentivirus for 2 days followed by total RNA extraction. The mRNA expressions of Pim1 and HER family were examined by RT-qPCR followed by normalization with *actin* expression. **(D)** MCF7 cells were transiently transfected with the indicated concentration of Flag-Pim1 expression vector for 3 days followed by preparation of total lysates. Protein expressions were examined by western blot using the indicated antibodies. Results were expressed as mean ± S.E.M. of three independent experiments. *: *p* < 0.05; **: *p* < 0.01 as compared with control group.

We next investigated the mechanism underlying Pim1 inhibitor-mediated anti-cancer activity in HER2-expressing breast cancer cells. As shown in Figure 5A, Supplementary Figure S2A, SMI-4a decreased HER2 and p-4E-BP1 protein expression in a dose-dependent manner in SkBr3 cells. The activity of HER2-downstream signaling ERK was also inhibited by SMI-4a. In addition to HER2, EGFR and HER3 protein expressions were attenuated by SMI-4a. Similar results were also observed in another HER2-positive BT474 breast cancer cell line (Figure 5B, Supplementary Figure S2B). In the RT-qPCR analysis, we found that SMI-4a reduced the mRNA levels of all members of HER family in a dose-dependent manner in both BT474 (Figure 6A) and SkBr3 cells (Figure 6B). Silence of Pim1 expression with two individual shRNAs also decreased the mRNA expression of HER2 in BT474 cells (Figure 6C). Conversely, overexpression of Pim1 also increased HER2 and HER3 expressions in MCF7 cells (Figure 6D). These results suggest that Pim1 inhibitors suppressed HER2 expression in HER2-expressing breast cancer cells through the transcriptional level.

### Pim1 Inhibitors Overcome Lapatinib Resistance Through Downregulation of HER Family Expression

**FIGURE 7:**
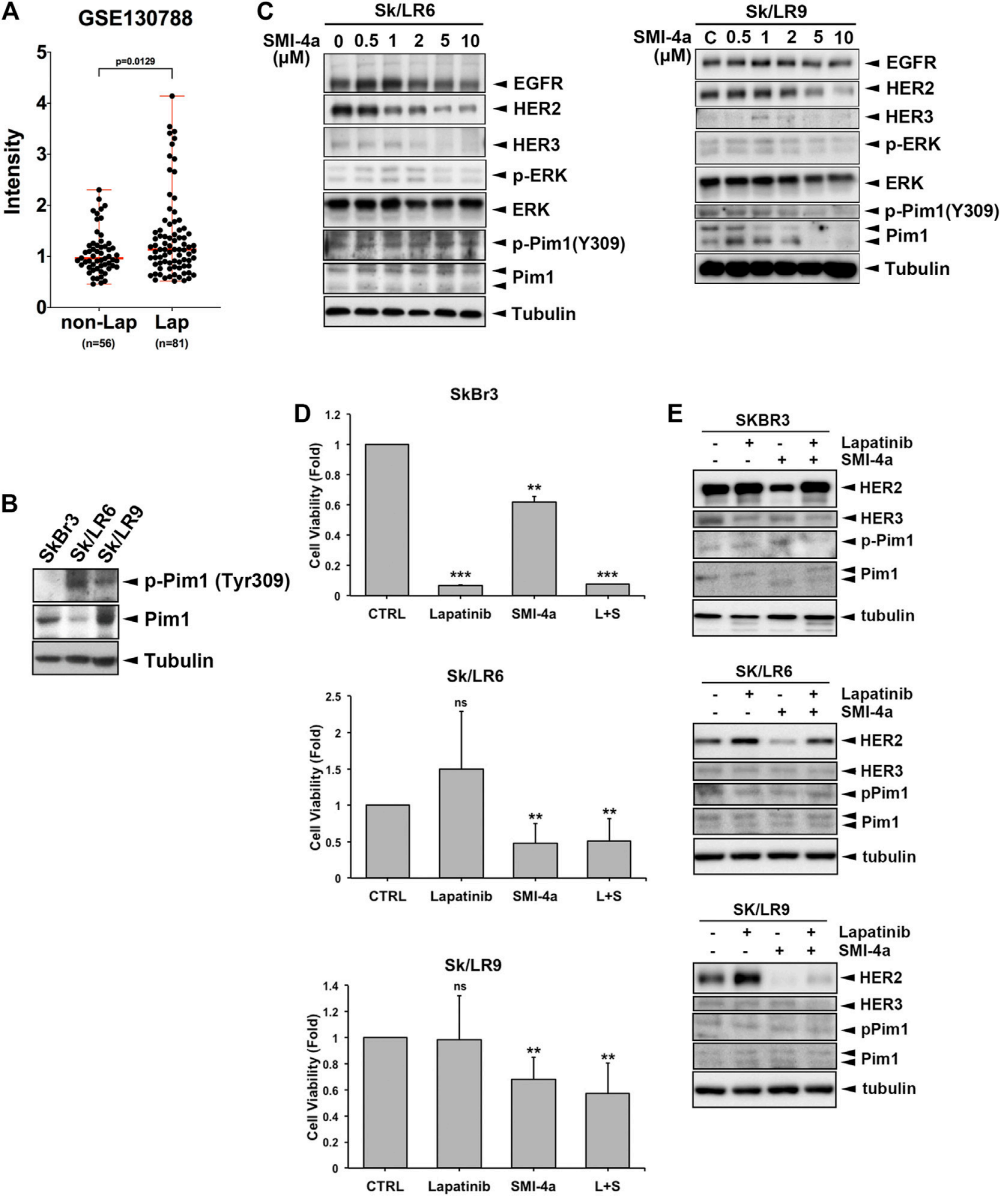
Pim1 inhibitor suppressed HER family expression and cell viability in lapatinib-resistant breast cancer cells. **(A)** The level of Pim1 mRNA in HER2-positive breast cancer patients who were treated with or without lapatinib in GEO database (GSE130788). **(B)** Total lysates of SkBr3 and its derived lapatinib-resistant Sk/LR6 and Sk/LR9 cells were harvested. Protein expressions were examined by western blot using indicated antibodies. **(C)** Sk/LR6 and Sk/LR9 cells were treated with SMI-4a at 0, 0.5, 1, 2, 5, 10 μM and whole-cell lysates were harvested. Protein expressions were examined by western blot using indicated antibodies. **(D)** SkBr3, Sk/LR6 and Sk/LR9 cells were treated with lapatinib (1 μM), SMI-4a (10 μM), the combination of lapatinib and SMI-4a, respectively for 3 days. Cell viability was examined by MTT assay. L + S: the combination of lapatinib and SMI-4a. Results were expressed as mean ± S.E.M. of three independent experiments. **: *p* < 0.01; ***: *p* < 0.001 as compared with control group. **(E)** SkBr3, Sk/LR6, and Sk/LR9 cells were treated with lapatinib (1 μM), SMI-4a (10 μM), the combination of lapatinib and SMI-4a, respectively for 3 days and whole-cell lysates were harvested. Protein expressions were examined by western blot using indicated antibodies.

Lapatinib is a HER2 TKI approved for metastatic HER2-positive breast cancer patients. Development of acquired resistance within one year of treatment limited the clinical benefits of this drug (D'amato et al., 2015; Shi et al., 2016). HER2 protein, even without tyrosine kinase activity in the presence of lapatinib, still contributes to the viability of lapatinib-resistant cells in a heregulin (HRG) and HER3-dependent manner (Sato et al., 2013). The tumoral Pim1 mRNA expression was higher in lapatinib-treated patients with HER2-positive breast cancers than in the patients without lapatinib treatment in a published gene set (GSE130788) (Figure 7A). Since Pim1 upregulates HER family expression, inhibition of HER family expression by Pim1 inhibitor may overcome lapatinib resistance. Interestingly, Sk/LR6 and Sk/LR9 cells, two acquired lapatinib-resistant clones of SkBr3 cells, exhibited higher Pim1 kinase activity as evidenced by the induction of Pim1 phosphorylation at Tyr309 than their parental SkBr3 cells (Figure 7B). When Sk/LR6 cells were treated with Pim1 inhibitor SMI-4a, the protein expressions of EGFR, HER2, HER3 as well as p-4E-BP1 were downregulated by Pim-1 inhibition in a dose-dependent manner (Figure 7C and Supplementary Figure S2C, D). We next examined whether SMI-4a overcomes lapatinib resistance in Sk/LR6 and Sk/LR9 cells. As shown in Figure 7D, treatment of SMI-4a, but not lapatinib, obviously inhibited cell viability of Sk/LR6 and Sk/LR9 cells rather than their parental cells. Meanwhile, corresponding blots showed that HER2 expression was suppressed by SMI-4a but not lapatinib in both resistant clones (Figure 7E). These results suggest that Pim1 inhibitor suppresses cell viability of lapatinib-resistant cells through reduction of HER2 expression.

## Discussion

The members of the HER family are well-known oncogenic driver genes in various cancer types. Although targeting the kinase activity by small molecular inhibitors has shown promising clinical benefits, kinase-independent functions have been proposed to contribute to the development of acquired resistance to these drugs (Zhang et al., 2009; Bhullar et al., 2018). Suppression of the protein expression of these RTKs has been proposed as a potential strategy to overcome the drug resistance (Bonanno et al., 2011; Alexander et al., 2017). In this study, we demonstrated that Pim1 may function as a therapeutic target to downregulate HER2 expression and thereby overcome lapatinib resistance.

Pim1 is a serine/threonine kinase and promotes cell proliferation, survival, and drug resistance. Overexpression of Pim1 has been observed in many cancer types and reported to play a crucial role in tumorigenesis (Narlik-Grassow et al., 2014; Warfel and Kraft, 2015). In previous studies, AKT inhibitor GSK690693 was reported to promote transcriptional induction of Pim1 kinase. Subsequently, Pim1 increased the RTK protein expression, including EGFR, HER2, and HER3 through Cap-independent translation, resulting in the resistance of prostate cancer cells to AKT inhibition (Cen et al., 2013). Moreover, Pim kinase inhibitor M-110 has been shown to reduce the expression of EGFR, leading to lower extracellular signal-regulated kinase (ERK) pathway activity in prostate cancer (Siu et al., 2011). Our results also demonstrated that Pim1 regulates protein expression of the HER family in breast cancer cells (Figures 5, 6), indicating that a common upregulation of the HER family by Pim-1 in various cancer types. Different to the findings in the previous studies, our data showed that Pim1 regulates the expression of HER family, in particular HER2, at the transcriptional level (Figure 6). It is known that Pim1 influences the activity of a number of transcriptional regulators, such as NFATc1, RelA/p65, and c-Myb (Rainio et al., 2002; Winn et al., 2003; Kim et al., 2010). Our previous study indicates that RelA/p65 activation mediates hepatitis B virus X protein-induced HER3 transcription (Chen et al., 2016). Furthermore, transcription factor activator protein-2 (AP-2) was reported to promote EGFR, HER2, and HER3 transcription (Bosher et al., 1995; Johnson, 1996; Bates and Hurst, 1997). Whether RelA/p65 or AP-2 is involved in Pim1-upregulated HER family expression awaits further investigations. In addition to transcriptional control, Pim1 was reported to promote cell cycle progression through induction of p27 phosphorylation and proteasomal degradation (Morishita et al., 2008). Therefore, the potential mechanisms other than transcriptional regulation for Pim1-mediated HER family expression cannot be excluded.

In breast cancer, 20–30% of cases belong to the subgroup of HER2 overexpression, which makes the tumor more aggressive. Therefore, targeted therapy against HER2 activity has been developed and approved for HER2-positive breast cancer (Iqbal and Iqbal, 2014). Although these drugs indeed showed clinical benefits to HER2-positive breast cancer patients, acquired resistance is developed eventually and becomes a hurdle to be overcome (Nahta et al., 2009; Rexer and Arteaga, 2012; D'amato et al., 2015; Pernas and Tolaney, 2019). To date, several mechanisms are proposed for lapatinib resistance. Upregulation of HRG has been observed in lapatinib-resistant cells to confer lapatinib resistance through HER3 and AKT activation, which depends on residual HER2 expression (Sato et al., 2013). In addition to its ligand upregulation, protein expression and phosphorylation of HER3 are induced by lapatinib. Phosphorylated HER3 is able to interact with the p85 subunit of PI3K to activate AKT signaling. Upregulated HER3 interacts with other RTK, such as MET, to maintain survival signaling (Sergina et al., 2007; Garrett et al., 2011; Chen et al., 2012). These events limit the therapeutic efficacy of lapatinib. Furthermore, HER2 T798I and EGFR T790M mutations have also been proposed to mediate lapatinib resistance (Trowe et al., 2008). On the other hand, accumulated evidence has revealed that EGFR promotes cancer cell survival through tyrosine kinase activity-independent mechanisms (Weihua et al., 2008; Tan et al., 2015; Tsuchihashi et al., 2016). Even its kinase activity is inhibited by lapatinib, EGFR still can confer survival signal in cancer cells. Therefore, targeting protein expression of the HER family rather than only its kinase activity may be an effective way for HER2-positive breast cancer cells. Indeed, our results showed that Pim1 inhibitors overcome lapatinib resistance by suppressing protein levels of the HER family (Figure 7). In addition, long-term treatment with lapatinib may switch oncogene addiction to the Pim1-regulated pathway, resulting in a stronger viability inhibition by SMI-4a in lapatinib-resistant clones. Moreover, these findings imply the existence of non-tyrosine phosphorylation-dependent functions of HER2, which may cause the drug resistance to lapatinib and need to be explored in further studies. In conclusion, our study indicates that downregulation of HER2 by targeting Pim1 may be a promising and effective therapeutic approach for HER2-positive breast cancer cells and for circumventing lapatinib resistance.

## Data Availability

The original contributions presented in the study are included in the article/Supplementary Material, further inquiries can be directed to the corresponding authors.

## References

[B1] Ahmed S. Sami A. Xiang J. (2015). HER2-directed Therapy: Current Treatment Options for HER2-Positive Breast Cancer. Breast Cancer 22, 101–116. 10.1007/s12282-015-0587-x 25634227

[B2] Alexander P. B. Chen R. Gong C. Yuan L. Jasper J. S. Ding Y. (2017). Distinct Receptor Tyrosine Kinase Subsets Mediate Anti-HER2 Drug Resistance in Breast Cancer. J. Biol. Chem. 292, 748–759. 10.1074/jbc.m116.754960 27903634 PMC5241747

[B3] Barros F. F. T. Powe D. G. Ellis I. O. Green A. R. (2010). Understanding the HER Family in Breast Cancer: Interaction with Ligands, Dimerization and Treatments. Histopathology 56, 560–572. 10.1111/j.1365-2559.2010.03494.x 20459566

[B4] Bates N. P. Hurst H. C. (1997). Transcriptional Regulation of Type I Receptor Tyrosine Kinases in the Mammary Gland. J. Mammary Gland Biol. Neoplasia 2, 153–163. 10.1023/a:1026303814855 10882301

[B5] Bhullar K. S. Lagaron N. O. Mcgowan E. M. Parmar I. Jha A. Hubbard B. P. (2018). Kinase-targeted Cancer Therapies: Progress, Challenges and Future Directions. Mol. Cancer 17, 48. 10.1186/s12943-018-0804-2 29455673 PMC5817855

[B6] Bonanno L. Jirillo A. Favaretto A. (2011). Mechanisms of Acquired Resistance to Epidermal Growth Factor Receptor Tyrosine Kinase Inhibitors and New Therapeutic Perspectives in Non Small Cell Lung Cancer. Cdt 12, 922–933. 10.2174/138945011795528958 21443472

[B7] Bosher J. M. Williams T. Hurst H. C. (1995). The Developmentally Regulated Transcription Factor AP-2 Is Involved in C-erbB-2 Overexpression in Human Mammary Carcinoma. Proc. Natl. Acad. Sci. 92, 744–747. 10.1073/pnas.92.3.744 7846046 PMC42696

[B8] Brandes A. A. Franceschi E. Tosoni A. Degli Esposti R. (2010). Trastuzumab and Lapatinib beyond Trastuzumab Progression for Metastatic Breast Cancer: Strategies and Pitfalls. Expert Rev. Anticancer Ther. 10, 179–184. 10.1586/era.09.156 20131994

[B9] Cen B. Mahajan S. Wang W. Kraft A. S. (2013). Elevation of Receptor Tyrosine Kinases by Small Molecule AKT Inhibitors in Prostate Cancer Is Mediated by Pim-1. Cancer Res. 73, 3402–3411. 10.1158/0008-5472.can-12-4619 23585456 PMC3680595

[B10] Chen C.-T. Kim H. Liska D. Gao S. Christensen J. G. Weiser M. R. (2012). MET Activation Mediates Resistance to Lapatinib Inhibition of HER2-Amplified Gastric Cancer Cells. Mol. Cancer Ther. 11, 660–669. 10.1158/1535-7163.mct-11-0754 22238368 PMC4209288

[B11] Chen J.-Y. Chen Y.-J. Yen C.-J. Chen W.-S. Huang W.-C. (2016). HBx Sensitizes Hepatocellular Carcinoma Cells to Lapatinib by Up-Regulating ErbB3. Oncotarget 7, 473–489. 10.18632/oncotarget.6337 26595522 PMC4808012

[B12] D'amato V. Raimondo L. Formisano L. Giuliano M. De Placido S. Rosa R. (2015). Mechanisms of Lapatinib Resistance in HER2-Driven Breast Cancer. Cancer Treat. Rev. 41, 877–883. 10.1016/j.ctrv.2015.08.001 26276735

[B13] Fahad Ullah M. (2019). Breast Cancer: Current Perspectives on the Disease Status. Adv. Exp. Med. Biol. 1152, 51–64. 10.1007/978-3-030-20301-6_4 31456179

[B14] Garrett J. T. Olivares M. G. Rinehart C. Granja-Ingram N. D. Sanchez V. Chakrabarty A. (2011). Transcriptional and Posttranslational Up-Regulation of HER3 (ErbB3) Compensates for Inhibition of the HER2 Tyrosine Kinase. Proc. Natl. Acad. Sci. 108, 5021–5026. 10.1073/pnas.1016140108 21385943 PMC3064360

[B15] Hicks M. Macrae E. R. Abdel‐Rasoul M. Layman R. Friedman S. Querry J. (2015). Neoadjuvant Dual HER2‐Targeted Therapy with Lapatinib and Trastuzumab Improves Pathologic Complete Response in Patients with Early Stage HER2‐Positive Breast Cancer: A Meta‐Analysis of Randomized Prospective Clinical Trials. The Oncologist 20, 337–343. 10.1634/theoncologist.2014-0334 25732265 PMC4391765

[B16] Holder S. Abdulkadir S. (2014). PIM1 Kinase as a Target in Prostate Cancer: Roles in Tumorigenesis, Castration Resistance, and Docetaxel Resistance. Ccdt 14, 105–114. 10.2174/1568009613666131126113854 24274399

[B17] Hsu J. L. Hung M.-C. (2016). The Role of HER2, EGFR, and Other Receptor Tyrosine Kinases in Breast Cancer. Cancer Metastasis Rev. 35, 575–588. 10.1007/s10555-016-9649-6 27913999 PMC5215954

[B18] Huang W.-C. Hung C.-M. Wei C.-T. Chen T.-M. Chien P.-H. Pan H.-L. (2016). Interleukin-6 Expression Contributes to Lapatinib Resistance through Maintenance of Stemness Property in HER2-Positive Breast Cancer Cells. Oncotarget 7, 62352–62363. 10.18632/oncotarget.11471 27694691 PMC5308732

[B19] Huang W. C. Hsieh Y. L. Hung C. M. Chien P. H. Chien Y. F. Chen L. C. (2013). BCRP/ABCG2 Inhibition Sensitizes Hepatocellular Carcinoma Cells to Sorafenib. PLoS One 8, e83627. 10.1371/journal.pone.0083627 24391798 PMC3877048

[B20] Iqbal N. Iqbal N. (2014). Human Epidermal Growth Factor Receptor 2 (HER2) in Cancers: Overexpression and Therapeutic Implications. Mol. Biol. Int. 2014, 852748. 10.1155/2014/852748 25276427 PMC4170925

[B21] Johnson A. C. (1996). Activation of Epidermal Growth Factor Receptor Gene Transcription by Phorbol 12-myristate 13-acetate Is Mediated by Activator Protein 2. J. Biol. Chem. 271, 3033–3038. 10.1016/s0021-9258(18)97974-3 8621697

[B22] Kim K. Kim J. H. Youn B. U. Jin H. M. Kim N. (2010). Pim-1 Regulates RANKL-Induced Osteoclastogenesis via NF-Κb Activation and NFATc1 Induction. J.I. 185, 7460–7466. 10.4049/jimmunol.1000885 21068407

[B23] Lee H.-P. Chen P.-C. Wang S.-W. Fong Y.-C. Tsai C.-H. Tsai F.-J. (2019a). Plumbagin Suppresses Endothelial Progenitor Cell-Related Angiogenesis *In Vitro* and *In Vivo* . J. Funct. Foods 52, 537–544. 10.1016/j.jff.2018.11.040

[B24] Lee H.-P. Wang S.-W. Wu Y.-C. Lin L.-W. Tsai F.-J. Yang J.-S. (2020). Soya-cerebroside Inhibits VEGF-Facilitated Angiogenesis in Endothelial Progenitor Cells. Food Agric. Immunol. 31, 193–204. 10.1080/09540105.2020.1713055

[B25] Lee H.-P. Wang S.-W. Wu Y.-C. Tsai C.-H. Tsai F.-J. Chung J.-G. (2019b). Glucocerebroside Reduces Endothelial Progenitor Cell-Induced Angiogenesis. Food Agric. Immunol. 30, 1033–1045. 10.1080/09540105.2019.1660623

[B26] Lin A. Giuliano C. J. Palladino A. John K. M. Abramowicz C. Yuan M. L. (2019). Off-target Toxicity Is a Common Mechanism of Action of Cancer Drugs Undergoing Clinical Trials. Sci. Transl Med. 11, eaaw8412. 10.1126/scitranslmed.aaw8412 31511426 PMC7717492

[B27] Luszczak S. Kumar C. Sathyadevan V. K. Simpson B. S. Gately K. A. Whitaker H. C. (2020). PIM Kinase Inhibition: Co-targeted Therapeutic Approaches in Prostate Cancer. Signal. Transduct Target. Ther. 5, 7. 10.1038/s41392-020-0109-y 32296034 PMC6992635

[B28] Morishita D. Katayama R. Sekimizu K. Tsuruo T. Fujita N. (2008). Pim Kinases Promote Cell Cycle Progression by Phosphorylating and Down-Regulating p27Kip1 at the Transcriptional and Posttranscriptional Levels. Cancer Res. 68, 5076–5085. 10.1158/0008-5472.can-08-0634 18593906

[B29] Nahta R. Shabaya S. Ozbay T. Rowe D. (2009). Personalizing HER2-Targeted Therapy in Metastatic Breast Cancer beyond HER2 Status: what We Have Learned from Clinical Specimens. Cppm 7, 263–274. 10.2174/187569209790112337 PMC2840656 20300449

[B30] Nami B. Wang Z. (2017). HER2 in Breast Cancer Stemness: A Negative Feedback Loop towards Trastuzumab Resistance. Cancers (Basel) 9, 40. 10.3390/cancers9050040 PMC5447950 28445439

[B31] Narlik-Grassow M. Blanco-Aparicio C. Carnero A. (2014). The PIM Family of Serine/threonine Kinases in Cancer. Med. Res. Rev. 34, 136–159. 10.1002/med.21284 23576269

[B32] Ouhtit A. Muzumdar S. Gupta I. Shanmuganathan S. Tamimi Y. (2015). Understanding the Functional Discrepancy of Pim-1 in Cancer. Front. Biosci. (Elite Ed. 7, 208–214. 10.2741/728 25553374

[B33] Pernas S. Tolaney S. M. (2019). HER2-positive Breast Cancer: New Therapeutic Frontiers and Overcoming Resistance. Ther. Adv. Med. Oncol. 11, 1758835919833519. 10.1177/1758835919833519 30911337 PMC6425535

[B34] Raica M. Jung I. Cimpean A. M. Suciu C. Muresan A. M. (2009). From Conventional Pathologic Diagnosis to the Molecular Classification of Breast Carcinoma: Are We Ready for the Change? Rom. J. Morphol. Embryol. 50, 5–13. 19221640

[B35] Rainio E.-M. Sandholm J. Koskinen P. J. (2002). Cutting Edge: Transcriptional Activity of NFATc1 Is Enhanced by the Pim-1 Kinase. J. Immunol. 168, 1524–1527. 10.4049/jimmunol.168.4.1524 11823475

[B36] Rexer B. N. Arteaga C. L. (2012). Intrinsic and Acquired Resistance to HER2-Targeted Therapies in HER2 Gene-Amplified Breast Cancer: Mechanisms and Clinical Implications. Crit. Rev. Oncog 17, 1–16. 10.1615/critrevoncog.v17.i1.20 22471661 PMC3394454

[B37] Riese D. J. 2nd Stern D. F. (1998). Specificity within the EGF family/ErbB Receptor Family Signaling Network. Bioessays 20, 41–48. 10.1002/(sici)1521-1878(199801)20:1<41::aid-bies7>3.0.co;2-v 9504046

[B38] Santa-Maria C. A. Nye L. Mutonga M. B. Jain S. Gradishar W. J. (2016). Management of Metastatic HER2-Positive Breast Cancer: Where Are We and where Do We Go from Here? Oncology (Williston Park) 30, 148–155. 26892151

[B39] Sato Y. Yashiro M. Takakura N. (2013). Heregulin Induces Resistance to Lapatinib-Mediated Growth Inhibition of HER2-Amplified Cancer Cells. Cancer Sci. 104, 1618–1625. 10.1111/cas.12290 24112719 PMC7653524

[B40] Sergina N. V. Rausch M. Wang D. Blair J. Hann B. Shokat K. M. (2007). Escape from HER-Family Tyrosine Kinase Inhibitor Therapy by the Kinase-Inactive HER3. Nature 445, 437–441. 10.1038/nature05474 17206155 PMC3025857

[B41] Shi H. Zhang W. Zhi Q. Jiang M. (2016). Lapatinib Resistance in HER2+ Cancers: Latest Findings and New Concepts on Molecular Mechanisms. Tumour Biol. 37, 15411–15431. 10.1007/s13277-016-5467-2 27726101

[B42] Siu A. Virtanen C. Jongstra J. (2011). PIM Kinase Isoform Specific Regulation of MIG6 Expression and EGFR Signaling in Prostate Cancer Cells. Oncotarget 2, 1134–1144. 10.18632/oncotarget.386 22193779 PMC3282072

[B43] Tan X. Thapa N. Sun Y. Anderson R. A. (2015). A Kinase-independent Role for EGF Receptor in Autophagy Initiation. Cell 160, 145–160. 10.1016/j.cell.2014.12.006 25594178 PMC4297316

[B44] Trowe T. Boukouvala S. Calkins K. Cutler R. E. Jr. Fong R. Funke R. (2008). EXEL-7647 Inhibits Mutant Forms of ErbB2 Associated with Lapatinib Resistance and Neoplastic Transformation. Clin. Cancer Res. 14, 2465–2475. 10.1158/1078-0432.ccr-07-4367 18413839

[B45] Tsuchihashi K. Okazaki S. Ohmura M. Ishikawa M. Sampetrean O. Onishi N. (2016). The EGF Receptor Promotes the Malignant Potential of Glioma by Regulating Amino Acid Transport System Xc(-). Cancer Res. 76, 2954–2963. 10.1158/0008-5472.can-15-2121 26980765 PMC4873328

[B46] Wang Z. (2017). ErbB Receptors and Cancer. Methods Mol. Biol. 1652, 3–35. 10.1007/978-1-4939-7219-7_1 28791631

[B47] Warfel N. A. Kraft A. S. (2015). PIM Kinase (And Akt) Biology and Signaling in Tumors. Pharmacol. Ther. 151, 41–49. 10.1016/j.pharmthera.2015.03.001 25749412 PMC4957637

[B48] Weihua Z. Tsan R. Huang W.-C. Wu Q. Chiu C.-H. Fidler I. J. (2008). Survival of Cancer Cells Is Maintained by EGFR Independent of its Kinase Activity. Cancer Cell 13, 385–393. 10.1016/j.ccr.2008.03.015 18455122 PMC2413063

[B49] Winn L. M. Lei W. Ness S. A. (2003). Pim-1 Phosphorylates the DNA Binding Domain of C-Myb. Cell Cycle 2, 258–262. 10.4161/cc.2.3.383 12734436

[B50] Zhang J. Yang P. L. Gray N. S. (2009). Targeting Cancer with Small Molecule Kinase Inhibitors. Nat. Rev. Cancer 9, 28–39. 10.1038/nrc2559 19104514 PMC12406740

