# Supplementary material for: Pim1 Kinase Inhibitors Exert Anti-Cancer Activity Against HER2-Positive Breast Cancer Cells Through Downregulation of HER2
Source: Front Pharmacol. 2021 Jun 29;12:614673. doi: 10.3389/fphar.2021.614673 (PMC8276059; doi:10.3389/fphar.2021.614673)
Supplement: Supplementary file 1 [file DataSheet1.PDF]

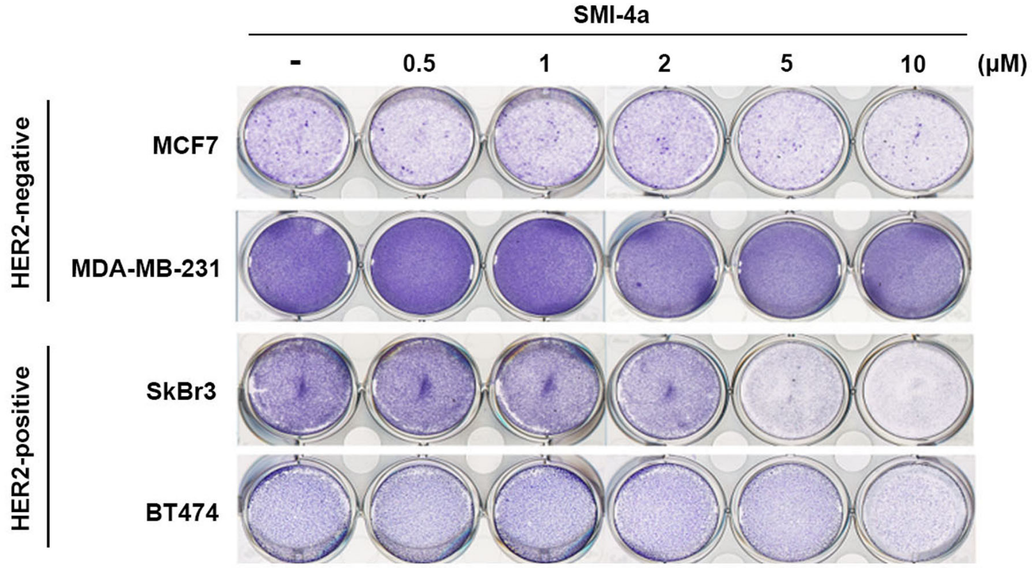

Supplementary Figure S1. HER2-expressing breast cancer cells were more sensitive to SMI-4a-induced inhibition of proliferation. MCF7, MDA-MB-231, SkBr3 and BT474 cells were treated with SMI-4a at 0, 0.5, 1, 2, 5, 10 μM and subjected to clonogenic assay for 14 days. Cell viability was determined by crystal violet staining.

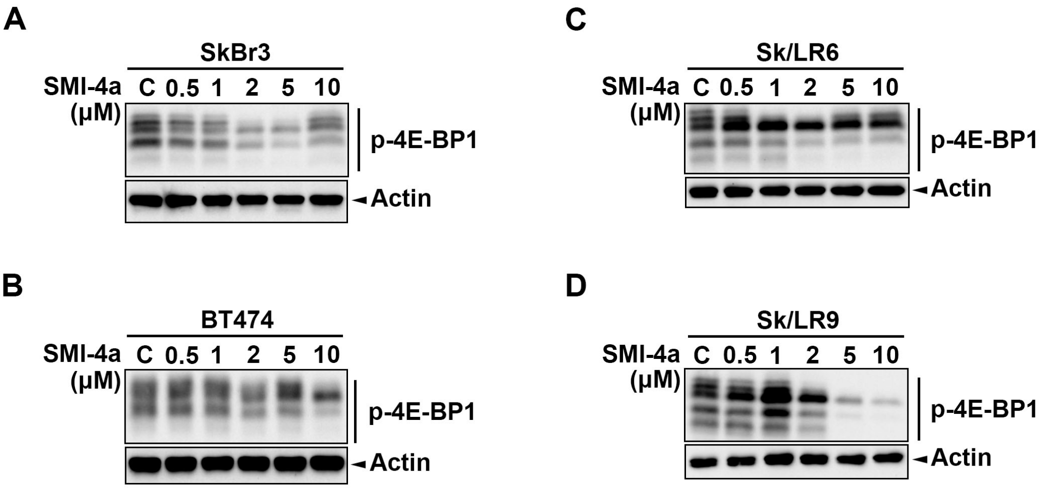

Supplementary Figure S2. SMI-4a suppressed Pim1 substrate p-4E-BP1 expression in different breast cancer cells. (A) SkBr3, (B) BT474, (C) Sk/LR6 and (D) Sk/LR9 cells were treated with SMI-4a at 0, 0.5, 1, 2, 5, 10 μM and whole cell lysates were harvested. Protein expressions were examined by western blot using indicated antibodies.
